# Real-World Prescribing Patterns and Clinical Characteristics of Patients With Type 2 Diabetes Mellitus Receiving Empagliflozin-Sitagliptin-Metformin Fixed-Dose Combination Therapy

**DOI:** 10.7759/cureus.115001

**Published:** 2026-08-22

**Authors:** Balaji T Naik, Shrimant Y, Debasish Patro, Suryakant Pankaj, Manoj Gaur, George Paulose, Rohan Kate, Dilip Gude, Ashish Patel, Sumit Sarkar, Aadil B Wasil, Utsa Basu, Ankit Chhabra, N Thyagarajan, Gurunath Chavan, Pradip Mate

**Affiliations:** 1 Endocrinology, Naik Endocrine and Diagnostic Centre, Nanded, IND; 2 Cardiology, Jayadeva Hospital, Bengaluru, IND; 3 Endocrinology, Endocrine and Diabetes Centre, Bhubaneswar, IND; 4 Medicine, Surya Health Clinic, Delhi, IND; 5 Cardiology, Devnishtha Heart Care Clinic, Delhi, IND; 6 General Medicine and Diabetology, Lisie Hospital, Kochi, IND; 7 Diabetology, Dr Kate Clinic, Pimpri-Chinchwad, IND; 8 Medicine, Yashoda Hospitals, Hyderabad, IND; 9 Medicine, Patel Medicine and Gynecology, Jabalpur, IND; 10 Medicine, Jyotsna Dental Clinic, Bardhaman, IND; 11 Cardiology, Ratsa Diagnostics, Kolkata, IND; 12 Diabetology, Dr. Utsa Basu Clinic, Kolkata, IND; 13 Endocrinology, R Gagan Multispeciality Hospital, Bathinda, IND; 14 Medicine, Krishna Clinic, Coimbatore, IND; 15 Medical Affairs, Lupin Limited, Mumbai, IND

**Keywords:** empagliflozin, fixed-dose combination, metformin, real-world evidence, sitagliptin, sodium-glucose transporter 2 inhibitors, type 2 diabetes mellitus

## Abstract

Background: Type 2 diabetes mellitus (T2DM) is associated with substantial cardiovascular and renal morbidity, necessitating individualized treatment strategies that target multiple pathophysiological mechanisms. Triple fixed-dose combination (FDC) therapy comprising empagliflozin, sitagliptin, and metformin (ESM) is used in routine clinical practice; however, real-world data describing the clinical characteristics and prescribing patterns of patients receiving this combination in India remain limited.

Methods: This multicentric, retrospective, cross-sectional real-world evidence study evaluated demographic characteristics, clinical profiles, comorbidities, glycemic parameters, renal function, and prescribing patterns among adult patients with T2DM receiving empagliflozin-, sitagliptin-, and metformin-based triple FDC therapy in India. Patients receiving empagliflozin 25 mg + sitagliptin 100 mg + metformin 1000 mg (ESM-25) or empagliflozin 10 mg + sitagliptin 100 mg + metformin 1000 mg (ESM-10) were included in the analysis.

Results: A total of 12,843 patients were included, comprising 7,060 receiving ESM-25 therapy and 5,783 receiving ESM-10 therapy. Most patients were aged >50 years (64.2% in both groups), and males constituted 60.4% and 61.2% of the ESM-25 and ESM-10 groups, respectively. Physicians and diabetologists accounted for the majority of prescriptions. Comorbidities were documented in the available medical records for 12.0% of patients, with hypertension and cardiovascular disease (CVD) being the most frequently recorded conditions. Patients receiving ESM-25 therapy demonstrated higher glycemic parameters than those receiving ESM-10 therapy, including HbA1c (8.80% vs 8.68%), fasting plasma glucose (182.38 mg/dL vs 163.96 mg/dL), and postprandial plasma glucose levels (247.99 mg/dL vs 216.54 mg/dL). Differences between dosage groups were observed for urinary albumin-to-creatinine ratio, LDL cholesterol, and triglyceride levels.

Conclusion: Empagliflozin-based triple FDC therapy was prescribed among Indian patients with T2DM, particularly older adults with cardiometabolic comorbidities. Distinct prescribing patterns and clinical characteristics observed between ESM-25 and ESM-10 therapy may reflect individualized treatment selection in routine clinical practice.

## Introduction

Type 2 diabetes mellitus (T2DM) is a major public health concern associated with substantial morbidity, mortality, and economic burden [1]. According to the International Diabetes Federation, approximately 590 million adults worldwide are living with diabetes, with India contributing substantially to the global disease burden [2]. Persistent hyperglycemia in T2DM is associated with progressive microvascular and macrovascular complications, including diabetic retinopathy, diabetic nephropathy, and diabetic neuropathy, as well as cardiovascular disease (CVD), cerebrovascular disease, and peripheral artery disease (PAD). These complications can contribute to chronic kidney disease (CKD), heart failure, stroke, and other adverse long-term outcomes. Persistent glucose elevation promotes oxidative stress, endothelial dysfunction, inflammation, and adverse cardiovascular remodeling, thereby accelerating cardiorenal damage and increasing the risk of long-term complications [3]. Consequently, early and effective glycemic control is essential to reduce diabetes-related complications and improve long-term outcomes [1].

The management of T2DM requires balancing optimal glycemic control with minimizing the risk of hypoglycemia, adverse effects, and treatment burden [4]. In addition to glycemic efficacy, factors such as cardiovascular and renal comorbidities, body weight, safety profile, treatment adherence, and patient-specific clinical characteristics play important roles in the selection of antidiabetic therapy. Current international guidelines, including the 2022 consensus report of the American Diabetes Association and European Association for the Study of Diabetes, recommend individualized treatment approaches with early initiation of combination therapy in patients inadequately controlled on monotherapy [2,4,5]. Fixed-dose combination (FDC) therapies involving agents with complementary mechanisms of action have therefore gained increasing importance in routine clinical practice [6]. In particular, triple-drug combinations incorporating metformin, sodium-glucose cotransporter-2 (SGLT2) inhibitors, and dipeptidyl peptidase-4 (DPP-4) inhibitors may provide improved glycemic control while simultaneously addressing associated cardiometabolic risk factors [7]. However, limited real-world evidence is available regarding the demographic and clinical characteristics of patients receiving empagliflozin-based triple FDC therapy in India.

Metformin remains the cornerstone of T2DM management because of its well-established efficacy, favorable safety profile, low risk of hypoglycemia, and beneficial metabolic effects [8]. Empagliflozin, an SGLT2 inhibitor, improves glycemic control by reducing renal glucose reabsorption and promoting urinary glucose excretion [9]. In addition to glycemic benefits, empagliflozin has demonstrated significant cardiovascular and renal protective effects, including reduction in cardiovascular mortality, hospitalization for heart failure, and progression of CKD [9,10]. Sitagliptin, a DPP-4 inhibitor, enhances incretin activity by inhibiting degradation of glucagon-like peptide-1 (GLP-1), thereby improving glucose-dependent insulin secretion and reducing glucagon levels. Sitagliptin is generally well tolerated and associated with a low risk of hypoglycemia and weight neutrality [11]. The combination of metformin, empagliflozin, and sitagliptin provides complementary glucose-lowering mechanisms targeting multiple pathophysiological defects involved in T2DM. The cardiovascular and renal benefits associated with empagliflozin are supported by evidence from SGLT2 inhibitor trials; however, these outcomes should not be attributed specifically to the ESM triple FDC in the absence of dedicated cardiovascular and renal outcome data for the combination [11]. Previous clinical and real-world studies have also demonstrated the efficacy and safety of empagliflozin-containing triple-drug regimens in patients with T2DM, supporting their use in routine clinical practice [12,13].

Despite the use of empagliflozin-based triple therapy in routine clinical practice in India, limited real-world evidence is available regarding the demographic characteristics, clinical profiles, comorbidity burden, and prescribing patterns associated with different dosage strengths of empagliflozin-containing FDC therapy. Understanding these real-world prescribing trends and patient characteristics is important for optimizing individualized treatment strategies in routine clinical practice. Therefore, this multicentric real-world evidence study aimed primarily to describe the demographic, clinical, comorbidity, laboratory, and prescribing characteristics of Indian patients with T2DM receiving empagliflozin-, sitagliptin-, and metformin-based triple fixed-dose combination (ESM-FDC) therapy. Secondary objectives were to descriptively compare these characteristics between patients receiving ESM-25 and ESM-10 and to explore clinical and prescribing characteristics across selected cardiovascular and renal comorbidity subgroups. These comparisons were exploratory and were not intended to assess comparative treatment efficacy, treatment effects, or clinical outcomes.

## Materials and methods

Study design

This was a multicentric, cross-sectional, retrospective, observational real-world evidence study conducted across multiple healthcare centers in India. The study period extended from November 12, 2025, to April 20, 2026. Retrospective medical records of adult patients with T2DM who had been prescribed ESM-FDC therapy in routine clinical practice were reviewed.

The study included patients from more than 300 healthcare centers across India. Individual center names and locations are not reported because of the large number of participating centers. Patients were identified from available medical records and case report forms (CRFs) and were assessed according to the predefined study eligibility criteria.

A total of 13,164 patients receiving ESM triple therapy were screened for inclusion in the study. After exclusion of patients with incomplete medical records, 12,843 patients were included in the final analysis, comprising 7,060 patients receiving ESM-25 therapy (empagliflozin 25 mg + sitagliptin 100 mg + metformin 1000 mg) and 5,783 patients receiving ESM-10 therapy (empagliflozin 10 mg + sitagliptin 100 mg + metformin 1000 mg). Cardiovascular and renal comorbidity subgroups were classified according to diagnoses documented in the available medical records/CRFs. These subgroup classifications were not mutually exclusive, and patients could therefore have more than one documented comorbidity.

Study population

The study population comprised adult male and female patients aged 18 years or older diagnosed with T2DM who had been prescribed ESM-FDC therapy according to the treating clinician’s discretion. Patients receiving either empagliflozin 25 mg + sitagliptin 100 mg + metformin 1000 mg or empagliflozin 10 mg + sitagliptin 100 mg + metformin 1000 mg were included in the analysis. Patients with type 1 diabetes mellitus, incomplete medical records, hypersensitivity to sitagliptin, SGLT2 inhibitors, or metformin, and those with known severe liver or kidney dysfunction (defined as eGFR < 30 mL/min/1.73 m²) at the time of treatment initiation were excluded from the study. Renal function was assessed using available eGFR data; however, the specific rationale for selecting ESM-10 versus ESM-25 was not recorded in the study.

Data collection

Data were retrospectively collected from available patient medical records using structured CRFs. Information collected included demographic characteristics such as age, gender, and body weight; healthcare provider specialty; glycemic parameters including glycated hemoglobin (HbA1c), fasting plasma glucose (FPG), postprandial plasma glucose (PPG), and random blood glucose (RBG); and documented comorbidities including hypertension, CVD, CKD, and PAD.

Comorbidities were considered present when the corresponding diagnosis was documented in the available patient medical record/CRF. No additional diagnostic screening or independent reassessment was performed for previously undocumented comorbidities. Cardiovascular disease history was captured as a categorical variable comprising coronary artery disease (CAD), ischemic heart disease (IHD), heart failure, valvular heart disease (VHD), stroke, or none. These specific cardiovascular disease history categories were mutually exclusive in the study database; however, the separately recorded CVD comorbidity variable was captured independently and could therefore differ from the specific cardiovascular disease history classification. Laboratory parameters, including serum creatinine, eGFR, urinary albumin-to-creatinine ratio (UACR), and lipid profile parameters, were recorded wherever available.

Because this was a retrospective study based on available medical records, a standardized patient-level index date corresponding to initiation of ESM-FDC therapy was not consistently available for all patients. Similarly, the exact timing of laboratory measurements relative to ESM prescription was not consistently documented. Therefore, a uniform predefined time window for classifying laboratory measurements as baseline could not be established. Laboratory and clinical parameters were analyzed when documented in the available medical records. Consequently, the term “baseline” in this study refers to the available clinical and laboratory information associated with the documented ESM prescription rather than measurements obtained within a predefined interval before or after treatment initiation.

Study outcomes

The primary objective was to describe the demographic, clinical, comorbidity, laboratory, and prescribing characteristics of patients with T2DM receiving ESM-FDC therapy.

Secondary objectives included descriptive comparison of these characteristics between patients receiving ESM-25 and ESM-10 and exploration of clinical and prescribing characteristics across selected cardiovascular and renal comorbidity subgroups. These comparisons were exploratory and were not intended to assess comparative treatment efficacy, treatment effects, treatment superiority, or clinical outcomes.

Statistical analysis

Statistical analysis was performed using IBM SPSS Statistics for Windows, Version 27.0 (Released 2019; IBM Corp., Armonk, NY, USA). Continuous variables were summarized as mean ± standard deviation (SD), whereas categorical variables were presented as frequencies and percentages. Between-group comparisons of continuous variables were performed using the independent-samples t-test, while categorical variables were compared using the Pearson chi-square test or Fisher’s exact test, as appropriate, based on the observed cell frequencies. Mean differences with corresponding 95% confidence intervals (CIs) and test statistics were calculated for continuous variables, while effect size estimates (Cohen's d) were calculated for the primary between-group comparison to quantify the magnitude of the observed difference. All statistical tests were two-tailed, and a p-value < 0.05 was considered statistically significant for descriptive purposes. Sample sizes varied across analyses because laboratory and clinical variables were available only for patients with documented measurements in the medical records. Missing data were handled using available-case analysis, and no imputation or substitution methods were applied. Subgroup analyses were considered exploratory because of the relatively small sample sizes for selected laboratory parameters. For subgroup analyses with very small numbers of available laboratory observations, findings were interpreted descriptively and p-values were considered potentially unstable. Given the large number of comparisons, no formal adjustment for multiple comparisons was performed; therefore, p-values from subgroup analyses were interpreted cautiously and considered hypothesis-generating, with emphasis placed on the magnitude and precision of between-group differences where available.

Ethical considerations

Ethical approval was obtained from the Central Independent Ethics Committee (CIEC), Pune, Maharashtra, India (IEC No. CIEC/2025/0337; approval dated 12 November 2025). As this was a multicentric retrospective real-world study involving investigators from multiple healthcare centers across India, ethical oversight was provided through the Central Independent Ethics Committee, which served as the independent ethics review board for the study. A waiver of informed consent was granted because the study involved retrospective analysis of de-identified patient data with minimal risk to participants. Patient confidentiality and data privacy were maintained throughout the study by anonymizing patient information prior to analysis.

## Results

A total of 13,164 patients were enrolled in the study, of whom 12,843 patients were included in the final analysis, and 321 patients were excluded because of incomplete data. Among the analyzed population, 7,060 patients received ESM-25 therapy (empagliflozin 25 mg + sitagliptin 100 mg + metformin 1000 mg), while 5,783 patients received ESM-10 therapy (empagliflozin 10 mg + sitagliptin 100 mg + metformin 1000 mg).

Demographic and clinical characteristics according to ESM triple therapy dosage

Table 1 presents the baseline demographic and clinical characteristics associated with the utilization of ESM triple therapy across dosage groups. Among patients receiving ESM-25 therapy, 64.2% were aged >50 years, 25.4% were aged 41-50 years, and 10.4% were aged 30-40 years. Similarly, among patients receiving ESM-10 therapy, 64.2% were aged >50 years, 25.8% were aged 41-50 years, and 10.1% were aged 30-40 years. Male patients accounted for 60.4% and 61.2% of patients receiving ESM-25 and ESM-10 therapy, respectively, whereas female patients accounted for 39.6% and 38.8%, respectively. Across healthcare specialties, physicians accounted for the highest proportion of ESM triple therapy prescriptions in both the ESM-25 (46.9%) and ESM-10 (47.2%) groups, followed by diabetologists, cardiologists, endocrinologists, and nephrologists.

**Table 1:** Demographic and clinical characteristics according to ESM triple therapy dosage

Variable	Category	ESM-25 (n = 7,060); n (%)	ESM-10 (n = 5,783); n (%)	Total (N = 12,843); n (%)
Age group	30-40 years	735 (10.4)	582 (10.1)	1,317 (10.3)
	41-50 years	1,793 (25.4)	1,490 (25.8)	3,283 (25.6)
	>50 years	4,532 (64.2)	3,711 (64.2)	8,243 (64.2)
Gender	Male	4,264 (60.4)	3,542 (61.2)	7,806 (60.8)
	Female	2,796 (39.6)	2,241 (38.8)	5,037 (39.2)
Healthcare provider specialty	Physician	3,308 (46.9)	2,728 (47.2)	6,036 (47.0)
	Diabetologist	2,182 (30.9)	1,559 (27.0)	3,741 (29.1)
	Cardiologist	798 (11.3)	1,058 (18.3)	1,856 (14.5)
	Endocrinologist	741 (10.5)	351 (6.1)	1,092 (8.5)
	Nephrologist	31 (0.4)	87 (1.5)	118 (0.9)
Comorbidity status	No comorbidity	6,241 (88.4)	5,060 (87.5)	11,301 (88.0)
	Comorbidity present	819 (11.6)	723 (12.5)	1,542 (12.0)
Comorbid conditions	Hypertension	429 (6.1)	368 (6.4)	797 (6.2)
	Cardiovascular disease	255 (3.6)	271 (4.7)	526 (4.1)
	Chronic kidney disease	76 (1.1)	69 (1.2)	145 (1.1)
	Peripheral artery disease	8 (0.1)	4 (0.1)	12 (0.1)
	Other comorbidities	51 (0.7)	11 (0.2)	62 (0.5)
Total		7,060 (100.0)	5,783 (100.0)	12,843 (100.0)

Comorbidities were identified from diagnoses documented in the available medical records and were not independently reassessed by the study investigator. Comorbidities were present in 11.6% of patients receiving ESM-25 therapy and 12.5% of those receiving ESM-10 therapy. Hypertension was the most frequently reported comorbid condition among patients receiving ESM-25 and ESM-10 therapy, observed in 6.1% and 6.4% of patients, respectively. CVD was reported in 3.6% and 4.7% of patients receiving ESM-25 and ESM-10 therapy, respectively, whereas CKD was reported in 1.1% and 1.2% of patients, respectively. PAD was reported in 0.1% of patients in both the ESM-25 and ESM-10 groups, whereas other comorbidities were reported in 0.7% and 0.2% of patients, respectively.

Anthropometric and laboratory characteristics according to ESM triple therapy dosage

Table 2 presents the baseline anthropometric, glycemic, renal function, and lipid profile parameters according to ESM triple therapy dosage groups. Patients receiving ESM-25 therapy had a mean (SD) body weight of 70.59 (11.83) kg, whereas those receiving ESM-10 therapy had a mean (SD) body weight of 69.78 (11.36) kg. The mean difference was 0.81 kg (95% CI: -0.06 to 1.67; Cohen's d = 0.069), indicating a negligible effect size, and the between-group difference was not statistically significant (t = 1.830, p = 0.067).

**Table 2:** Anthropometric and laboratory parameters according to ESM triple therapy dosage ESM: empagliflozin, sitagliptin, and metformin, RBG: random blood glucose, FPG: fasting plasma glucose, PPG: postprandial plasma glucose, eGFR: estimated glomerular filtration rate, UACR: urinary albumin-to-creatinine ratio, LDL: low-density lipoprotein, HDL: high-density lipoprotein.

Parameter	ESM-25; mean ± SD (n = 1,710)	ESM-10; mean ± SD (n = 1,177)	95% CI of mean difference	t	df	p-value	Cohen's d
Anthropometric parameter							
Weight (kg)	70.59 ± 11.83	69.78 ± 11.36	-0.06 to 1.67	1.830	2,885	0.067	0.069
Glycemic parameters							
HbA1c (%)	8.80 ± 1.51	8.68 ± 1.54	0.02-0.22	2.261	3,321	0.024	0.079
RBG (mg/dL)	236.03 ± 77.70	230.12 ± 73.05	-4.45 to 16.26	1.119	819	0.263	0.078
FPG (mg/dL)	182.38 ± 62.99	163.96 ± 53.60	12.02-24.80	5.654	1,357	<0.001	0.311
PPG (mg/dL)	247.99 ± 83.92	216.54 ± 78.48	22.65-40.27	7.004	1,343	<0.001	0.385
Renal function parameters							
Serum creatinine (mg/dL)	1.29 ± 0.70	1.31 ± 0.56	-0.14 to 0.09	-0.455	504	0.649	-0.040
eGFR (mL/min/1.73 m²)	69.24 ± 32.89	65.70 ± 22.71	-1.40 to 8.49	1.408	506	0.160	0.125
UACR (mg/g)	27.07 ± 76.19	11.27 ± 19.75	5.47-26.13	3.007	438	0.003	0.287
Lipid profile parameters							
HDL cholesterol (mg/dL)	42.75 ± 7.32	43.29 ± 6.04	-2.42 to 1.35	-0.558	202	0.577	-0.079
LDL cholesterol (mg/dL)	136.80 ± 57.04	172.79 ± 64.69	-46.20 to -25.80	-6.930	548	<0.001	-0.591
Triglycerides (mg/dL)	203.14 ± 64.44	213.92 ± 47.17	-20.29 to -1.28	-2.228	550	0.026	-0.190

Mean (SD) HbA1c levels were 8.80 (1.51)% in the ESM-25 group and 8.68 (1.54)% in the ESM-10 group. The mean difference was 0.12 percentage points (95% CI: 0.02-0.22; Cohen's d = 0.079), representing a very small effect size despite statistical significance (t = 2.261, p = 0.024). Mean (SD) RBG levels were 236.03 (77.70) mg/dL and 230.12 (73.05) mg/dL in the ESM-25 and ESM-10 groups, respectively, with no significant between-group difference (mean difference 5.91 mg/dL; 95% CI: -4.45 to 16.26; Cohen's d = 0.078; t = 1.119, p = 0.263). Mean (SD) FPG levels were 182.38 (62.99) mg/dL in the ESM-25 group and 163.96 (53.60) mg/dL in the ESM-10 group, corresponding to a mean difference of 18.41 mg/dL (95% CI: 12.02-24.80; Cohen's d = 0.311), representing a small-to-moderate effect size (t = 5.654, p < 0.001). Similarly, mean (SD) PPG levels were 247.99 (83.92) mg/dL in the ESM-25 group and 216.54 (78.48) mg/dL in the ESM-10 group, with a mean difference of 31.46 mg/dL (95% CI: 22.65-40.27; Cohen's d = 0.385), indicating a moderate effect size (t = 7.004, p < 0.001).

Mean (SD) serum creatinine levels were 1.29 (0.70) mg/dL in the ESM-25 group and 1.31 (0.56) mg/dL in the ESM-10 group. Mean (SD) eGFR values were 69.24 (32.89) mL/min/1.73 m² and 65.70 (22.71) mL/min/1.73 m² in the ESM-25 and ESM-10 groups, respectively. Mean (SD) UACR values were 27.07 (76.19) mg/g in the ESM-25 group and 11.27 (19.75) mg/g in the ESM-10 group. The mean difference in UACR was 15.80 mg/g (95% CI: 5.47-26.13; Cohen's d = 0.287), indicating a small effect size, and was statistically significant (t = 3.007, p = 0.003). Serum creatinine and eGFR were comparable between the two treatment groups.

Patients receiving ESM-25 therapy had mean (SD) HDL cholesterol, LDL cholesterol, and triglyceride levels of 42.75 (7.32) mg/dL, 136.80 (57.04) mg/dL, and 203.14 (64.44) mg/dL, respectively, compared with 43.29 (6.04) mg/dL, 172.79 (64.69) mg/dL, and 213.92 (47.17) mg/dL, respectively, in the ESM-10 group. LDL cholesterol showed the largest between-group difference (mean difference -36.00 mg/dL; 95% CI: -46.20 to -25.80; Cohen's d = -0.591; p < 0.001), whereas triglycerides demonstrated a smaller difference (mean difference -10.78 mg/dL; 95% CI: -20.29 to -1.28; Cohen's d = -0.190; p = 0.026). HDL cholesterol levels were comparable between the groups (p = 0.577).

Utilization of ESM triple therapy in patients with hypertension

Table 3 presents baseline clinical, biochemical, and cardiovascular characteristics associated with utilization of ESM triple therapy among hypertensive patients. Among patients receiving ESM-25 therapy, 65.7% were aged >50 years, whereas 69.0% of patients receiving ESM-10 therapy belonged to the same age category. Male patients accounted for 62.5% and 64.7% of patients receiving ESM-25 and ESM-10 therapy, respectively. Among healthcare provider specialties, diabetologists accounted for the highest proportion of ESM-25 prescriptions (38.0%), whereas physicians accounted for the highest proportion of ESM-10 prescriptions (50.0%). Healthcare provider specialty differed between the ESM-25 and ESM-10 groups, with diabetologists accounting for a higher proportion of ESM-25 prescriptions and physicians accounting for a higher proportion of ESM-10 prescriptions. Among cardiovascular comorbidities, CAD was reported in 33.6% of patients receiving ESM-25 therapy and 50.5% of patients receiving ESM-10 therapy. Heart failure was reported in 19.6% and 11.4% of patients receiving ESM-25 and ESM-10 therapy, respectively.

**Table 3:** Clinical characteristics of hypertensive patients receiving ESM triple therapy ESM: empagliflozin, sitagliptin, and metformin, CAD: coronary artery disease, IHD: ischemic heart disease, VHD: valvular heart disease, RBG: random blood glucose, FPG: fasting plasma glucose, PPG: postprandial plasma glucose, eGFR: estimated glomerular filtration rate, UACR: urinary albumin-to-creatinine ratio, HDL: high-density lipoprotein, LDL: low-density lipoprotein.

Parameter	ESM-25 (n = 429)	ESM-10 (n = 368)	Total	Statistical test	df	p-value
Age group, n (%)						
30-40 years	50 (11.7)	34 (9.2)	84 (10.5)	χ² = 1.483	2	0.476
41-50 years	97 (22.6)	80 (21.7)	177 (22.2)			
>50 years	282 (65.7)	254 (69.0)	536 (67.3)			
Healthcare provider specialty, n (%)						
Cardiologist	61 (14.2)	63 (17.1)	124 (15.6)	χ² = 63.971	4	<0.001
Diabetologist	163 (38.0)	69 (18.8)	232 (29.1)			
Endocrinologist	94 (21.9)	52 (14.1)	146 (18.3)			
Nephrologist	0 (0.0)	0 (0.0)	0 (0.0)			
Physician	111 (25.9)	184 (50.0)	295 (37.0)			
Gender, n (%)						
Male	268 (62.5)	238 (64.7)	506 (63.5)	χ² = 0.415	1	0.520
Female	161 (37.5)	130 (35.3)	291 (36.5)			
History of cardiovascular disease, n (%)						
CAD	144 (33.6)	186 (50.5)	330 (41.4)	χ² = 30.646	5	<0.001
IHD	11 (2.6)	14 (3.8)	25 (3.1)			
Heart failure	84 (19.6)	42 (11.4)	126 (15.8)			
VHD	7 (1.6)	2 (0.5)	9 (1.1)			
Stroke	2 (0.5)	0 (0.0)	2 (0.3)			
None	181 (42.2)	124 (33.7)	305 (38.3)			
Demographic parameters, mean ± SD						
Age (years)	55.07 ± 8.63	55.03 ± 9.06	55.05 ± 8.84	t = 0.064	795	0.949
Weight (kg)	73.86 ± 10.87	72.83 ± 10.90	73.38 ± 10.88	t = 1.068	512	0.286
Glycemic parameters, mean ± SD						
HbA1c (%)	9.13 ± 1.92	8.70 ± 1.32	8.92 ± 1.65	t = 2.780	466	0.006
RBG (mg/dL)	197.59 ± 57.24	230.40 ± 61.59	215.86 ± 59.42	t = -4.511	271	<0.001
FPG (mg/dL)	145.47 ± 38.13	143.49 ± 25.85	144.62 ± 32.84	t = 0.580	388	0.562
PPG (mg/dL)	208.05 ± 47.25	196.46 ± 38.54	202.99 ± 43.33	t = 2.585	384	0.010
Renal parameters, mean ± SD						
Serum creatinine (mg/dL)	1.34 ± 0.32	1.37 ± 0.64	1.35 ± 0.48	t = -0.496	300	0.620
eGFR (mL/min/1.73 m²)	61.74 ± 22.28	62.77 ± 21.75	62.21 ± 22.01	t = -0.405	301	0.686
UACR (mg/g)	18.22 ± 64.42	10.55 ± 20.92	14.66 ± 47.87	t = 1.304	280	0.193
Lipid parameters, Mean ± SD						
HDL cholesterol (mg/dL)	40.79 ± 6.87	43.29 ± 6.02	41.95 ± 6.58	t = -1.738	80	0.086
LDL cholesterol (mg/dL)	156.38 ± 47.62	167.11 ± 53.10	161.22 ± 50.32	t = -1.900	317	0.058
Triglycerides (mg/dL)	217.04 ± 48.39	210.63 ± 44.77	214.12 ± 46.80	t = 1.213	314	0.226

Mean (SD) age was 55.07 (8.63) years in the ESM-25 group and 55.03 (9.06) years in the ESM-10 group. Mean (SD) body weight was 73.86 (10.87) kg and 72.83 (10.90) kg in the ESM-25 and ESM-10 groups, respectively. Utilization of ESM-25 therapy in hypertensive patients was associated with mean (SD) HbA1c, FPG, and PPG levels of 9.13 (1.92)%, 145.47 (38.13) mg/dL, and 208.05 (47.25) mg/dL, respectively. Utilization of ESM-10 therapy was associated with mean (SD) HbA1c, FPG, and PPG levels of 8.70 (1.32)%, 143.49 (25.85) mg/dL, and 196.46 (38.54) mg/dL, respectively.

Statistically significant differences between ESM-25 and ESM-10 therapy were observed for HbA1c (p = 0.006), RBG (p < 0.0001), and PPG (p = 0.010), whereas FPG, serum creatinine, eGFR, UACR, and lipid parameters were comparable between the two dosage groups.

Utilization of ESM triple therapy in patients with CVD

Table 4 presents baseline clinical, biochemical, and cardiovascular characteristics associated with utilization of ESM triple therapy among patients with CVD. Among patients receiving ESM-25 therapy, 64.3% were aged >50 years, whereas 59.8% of patients receiving ESM-10 therapy belonged to the same age category. Male patients accounted for 57.3% and 69.7% of patients receiving ESM-25 and ESM-10 therapy, respectively. Among healthcare provider specialties, diabetologists accounted for the highest proportion of ESM-25 prescriptions (44.7%), whereas cardiologists accounted for the highest proportion of ESM-10 prescriptions (36.9%). A statistically significant association was observed between healthcare provider specialty and ESM triple therapy dosage groups (p < 0.0001). Among cardiovascular disease histories, heart failure was reported in 38.4% and 48.7% of patients receiving ESM-25 and ESM-10 therapy, respectively, whereas CAD was reported in 40.0% and 36.2% of patients, respectively.

**Table 4:** Clinical characteristics of patients with cardiovascular disease receiving ESM triple therapy ESM: empagliflozin, sitagliptin, and metformin, CAD: coronary artery disease, IHD: ischemic heart disease, VHD: valvular heart disease, RBG: random blood glucose, FPG: fasting plasma glucose, PPG: postprandial plasma glucose, eGFR: estimated glomerular filtration rate, UACR: urinary albumin-to-creatinine ratio, HDL: high-density lipoprotein, LDL: low-density lipoprotein

Parameter	ESM-25 (n = 255)	ESM-10 (n = 271)	Statistical test	df	p-value
Age group, n (%)					
30-40 years	21 (8.2)	30 (11.1)	χ² = 1.659	2	0.436
41-50 years	70 (27.5)	79 (29.2)			
>50 years	164 (64.3)	162 (59.8)			
Healthcare provider specialty, n (%)					
Cardiologist	48 (18.8)	100 (36.9)	χ² = 69.726	4	<0.001
Diabetologist	114 (44.7)	39 (14.4)			
Endocrinologist	43 (16.9)	36 (13.3)			
Nephrologist	0 (0.0)	0 (0.0)			
Physician	50 (19.6)	96 (35.4)			
Gender, n (%)					
Male	146 (57.3)	189 (69.7)	χ² = 8.858	1	0.003
Female	109 (42.7)	82 (30.3)			
History of cardiovascular disease, n (%)					
CAD	102 (40.0)	98 (36.2)	χ² = 8.511	5	0.075
IHD	16 (6.3)	7 (2.6)			
Heart failure	98 (38.4)	132 (48.7)			
VHD	2 (0.8)	2 (0.7)			
Stroke	0 (0.0)	0 (0.0)			
None	37 (14.5)	32 (11.8)			
Demographic parameters					
Age (years), mean ± SD	53.91 ± 9.87	54.07 ± 9.78	t = -0.183	524	0.855
Weight (kg), mean ± SD	67.62 ± 12.67	69.05 ± 12.89	t = -0.964	299	0.336
Glycemic parameters					
HbA1c (%), mean ± SD	8.53 ± 1.41	8.62 ± 1.33	t = -0.606	342	0.545
RBG (mg/dL), mean ± SD	240.66 ± 95.19	202.79 ± 42.08	t = 3.258	143	0.001
FPG (mg/dL), mean ± SD	162.65 ± 34.82	159.21 ± 27.09	t = 1.005	324	0.316
PPG (mg/dL), mean ± SD	249.38 ± 64.37	222.45 ± 51.27	t = 4.079	305	<0.001
Renal parameters					
Serum creatinine (mg/dL), mean ± SD	1.61 ± 1.59	1.29 ± 0.42	t = 1.701	118	0.092
eGFR (mL/min/1.73 m²), mean ± SD	58.41 ± 33.12	65.97 ± 21.70	t = -1.492	118	0.138
UACR (mg/g), Mean ± SD	11.34 ± 12.37	13.33 ± 19.11	t = −0.554	110	0.581
Lipid parameters					
HDL cholesterol (mg/dL), mean ± SD	47.95 ± 7.49	41.27 ± 5.33	t = 4.045	60	<0.001
LDL cholesterol (mg/dL), mean ± SD	115.19 ± 74.97	192.06 ± 73.52	t = -5.862	136	<0.001
Triglycerides (mg/dL), mean ± SD	211.52 ± 65.94	223.81 ± 43.79	t = -1.305	135	0.194

Mean (SD) age was 53.91 (9.87) years in the ESM-25 group and 54.07 (9.78) years in the ESM-10 group. Mean (SD) body weight was 67.62 (12.67) kg and 69.05 (12.89) kg in the ESM-25 and ESM-10 groups, respectively. Utilization of ESM-25 therapy in patients with CVD was associated with mean (SD) HbA1c, FPG, and PPG levels of 8.53 (1.41)%, 162.65 (34.82) mg/dL, and 249.38 (64.37) mg/dL, respectively. Utilization of ESM-10 therapy was associated with mean (SD) HbA1c, FPG, and PPG levels of 8.62 (1.33)%, 159.21 (27.09) mg/dL, and 222.45 (51.27) mg/dL, respectively.

Differences between the ESM-25 and ESM-10 groups were observed for healthcare provider specialty, gender distribution, RBG, PPG, HDL cholesterol, and LDL cholesterol, whereas HbA1c, FPG, serum creatinine, eGFR, UACR, and triglyceride levels were comparable between the two dosage groups.

Utilization of ESM triple therapy in patients with CKD

Table 5 presents baseline clinical, biochemical, and cardiovascular characteristics associated with utilization of ESM triple therapy among patients with CKD. Among patients receiving ESM-25 therapy, 65.8% were aged >50 years, whereas 69.6% of those receiving ESM-10 therapy were in the same age category. Male patients accounted for 55.3% and 69.6% of patients receiving ESM-25 and ESM-10 therapy, respectively. Among healthcare provider specialties, diabetologists accounted for the highest proportion of ESM triple therapy prescriptions in both the ESM-25 (56.6%) and ESM-10 (47.8%) groups. No statistically significant association was observed between healthcare provider specialty and ESM triple therapy dosage groups (p = 0.347). Among CVD histories, heart failure was reported in 26.3% and 30.4% of patients receiving ESM-25 and ESM-10 therapy, respectively, whereas CAD was reported in 18.4% and 11.6% of patients, respectively.

**Table 5:** Clinical characteristics of patients with CKD receiving ESM triple therapy ESM: empagliflozin, sitagliptin, and metformin, CAD: coronary artery disease, IHD: ischemic heart disease, VHD: valvular heart disease, RBG: random blood glucose, FPG: fasting plasma glucose, PPG: postprandial plasma glucose, eGFR: estimated glomerular filtration rate, UACR: urinary albumin-to-creatinine ratio, HDL: high-density lipoprotein, LDL: low-density lipoprotein.

Parameter	ESM-25 (n = 76)	ESM-10 (n = 69)	Statistical test	df	p-value
Age group, n (%)					
30-40 years	7 (9.2)	7 (10.1)	χ² = 0.462	2	0.794
41-50 years	19 (25.0)	14 (20.3)			
>50 years	50 (65.8)	48 (69.6)			
Healthcare provider specialty, n (%)					
Cardiologist	23 (30.3)	23 (33.3)	χ² = 3.301	4	0.347
Diabetologist	43 (56.6)	33 (47.8)			
Endocrinologist	3 (3.9)	1 (1.4)			
Nephrologist	0 (0.0)	0 (0.0)			
Physician	7 (9.2)	12 (17.4)			
Gender, n (%)					
Male	42 (55.3)	48 (69.6)	χ² = 3.142	1	0.076
Female	34 (44.7)	21 (30.4)			
History of cardiovascular disease, n (%)					
CAD	14 (18.4)	8 (11.6)	χ² = 4.347	5	0.501
IHD	2 (2.6)	2 (2.9)			
Heart failure	20 (26.3)	21 (30.4)			
VHD	3 (3.9)	0 (0.0)			
Stroke	1 (1.3)	1 (1.4)			
None	36 (47.4)	37 (53.6)			
Demographic parameters					
Age (years), Mean ± SD	56.92 ± 10.72	54.09 ± 10.27	t = 1.621	143	0.107
Weight (kg), Mean ± SD	70.84 ± 10.75	72.38 ± 11.05	t = -0.484	45	0.631
Glycemic parameters					
HbA1c (%), mean ± SD	8.29 ± 0.91	8.91 ± 1.15	t = -2.102	47	0.041
RBG (mg/dL), mean ± SD	303.15 ± 87.52	251.50 ± 40.01	t = 2.181	34	0.036
FPG (mg/dL), mean ± SD	179.05 ± 40.02	159.13 ± 18.11	t = 1.795	34	0.082
PPG (mg/dL), mean ± SD	276.30 ± 76.93	218.60 ± 30.91	t = 2.736	33	0.010
Renal parameters					
Serum creatinine (mg/dL), mean ± SD	1.04 ± 0.11	1.47 ± 0.20	t = -4.408	14	0.001
eGFR (mL/min/1.73 m²), mean ± SD	66.00 ± 26.94	53.36 ± 7.86	t = 1.479	15	0.160
UACR (mg/g), Mean ± SD	18.36 ± 12.07	1.37 ± 0.14	t = 5.015	13	<0.001
Lipid parameters					
HDL cholesterol (mg/dL), mean ± SD	41.20 ± 3.35				
LDL cholesterol (mg/dL), mean ± SD	121.17 ± 53.54	248.64 ± 20.79	t = -7.122	15	<0.001
Triglycerides (mg/dL), mean ± SD	187.83 ± 23.95	254.91 ± 29.36	t = -4.775	15	<0.001

Mean (SD) age was 56.92 (10.72) years in the ESM-25 group and 54.09 (10.27) years in the ESM-10 group. Mean (SD) body weight was 70.84 (10.75) kg and 72.38 (11.05) kg in the ESM-25 and ESM-10 groups, respectively.

Utilization of ESM-25 therapy in patients with CKD was associated with mean (SD) HbA1c, FPG, and PPG levels of 8.29 (0.91)%, 179.05 (40.02) mg/dL, and 276.30 (76.93) mg/dL, respectively. Utilization of ESM-10 therapy was associated with mean (SD) HbA1c, FPG, and PPG levels of 8.91 (1.15)%, 159.13 (18.11) mg/dL, and 218.60 (30.91) mg/dL, respectively.

Statistically significant differences between ESM-25 and ESM-10 therapy were observed for HbA1c (p = 0.041), RBG (p = 0.036), PPG (p = 0.010), serum creatinine (p = 0.001), UACR (p < 0.0001), LDL cholesterol (p < 0.0001), and triglyceride levels (p < 0.0001); these statistical findings should be interpreted in conjunction with the magnitude of the corresponding between-group differences. Age, body weight, FPG, eGFR, and healthcare provider specialty distribution were comparable between the two dosage groups.

Utilization of ESM triple therapy in patients with CAD

Table 6 presents the clinical and biochemical characteristics associated with utilization of ESM triple therapy among patients with CAD. Among patients receiving ESM-25 therapy, 66.1% were aged >50 years, whereas 68.6% of patients receiving ESM-10 therapy belonged to the same age category. Male patients accounted for 64.0% and 66.0% of patients receiving ESM-25 and ESM-10 therapy, respectively. Among healthcare provider specialties, diabetologists accounted for the highest proportion of ESM-25 prescriptions (33.9%), whereas physicians accounted for the highest proportion of ESM-10 prescriptions (41.3%). A statistically significant association was observed between healthcare provider specialty and ESM triple therapy dosage groups (p < 0.0001).

**Table 6:** Clinical characteristics of patients with CAD receiving ESM triple therapy ESM: empagliflozin, sitagliptin, and metformin, CAD: coronary artery disease, IHD: ischemic heart disease, VHD: valvular heart disease, CVD: cardiovascular disease, CKD: chronic kidney disease, PAD: peripheral artery disease, RBG: random blood glucose, FPG: fasting plasma glucose, PPG: postprandial plasma glucose, eGFR: estimated glomerular filtration rate, UACR: urinary albumin-to-creatinine ratio, HDL: high-density lipoprotein, LDL: low-density lipoprotein.

Parameter	ESM-25 (n = 289)	ESM-10 (n = 312)	Statistical test	df	p-value
Age group, n (%)					
30-40 years	33 (11.4)	28 (9.0)	χ² = 1.022	2	0.600
41-50 years	65 (22.5)	70 (22.4)			
>50 years	191 (66.1)	214 (68.6)			
Healthcare provider specialty, n (%)					
Cardiologist	66 (22.8)	87 (27.9)	χ² = 36.397	4	<0.001
Diabetologist	98 (33.9)	46 (14.7)			
Endocrinologist	52 (18.0)	50 (16.0)			
Nephrologist	0 (0.0)	0 (0.0)			
Physician	73 (25.3)	129 (41.3)			
Gender, n (%)					
Male	185 (64.0)	206 (66.0)	χ² = 0.267	1	0.605
Female	104 (36.0)	106 (34.0)			
Comorbidities, n (%)					
Hypertension	144 (49.8)	186 (59.6)	χ² = 18.615	5	0.002
CVD	102 (35.3)	98 (31.4)			
CKD	14 (4.8)	8 (2.6)			
PAD	2 (0.7)	1 (0.3)			
Other	14 (4.8)	1 (0.3)			
None	13 (4.5)	18 (5.8)			
Demographic parameters, mean ± SD					
Age (years)	55.55 ± 10.09	55.17 ± 8.97	t = 0.480	599	0.631
Weight (kg)	71.69 ± 11.86	72.00 ± 11.25	t = -0.278	430	0.781
Glycemic parameters, mean ± SD					
HbA1c (%)	9.47 ± 2.24	9.05 ± 1.43	t = 2.231	391	0.026
RBG (mg/dL)	204.62 ± 67.01	229.68 ± 57.02	t = -3.350	273	0.001
FPG (mg/dL)	160.12 ± 41.91	159.61 ± 25.48	t = 0.123	286	0.903
PPG (mg/dL)	223.46 ± 65.31	209.35 ± 49.33	t = 2.026	277	0.044
Renal parameters, mean ± SD					
Serum creatinine (mg/dL)	1.38 ± 1.09	1.25 ± 0.27	t = 1.146	185	0.253
eGFR (mL/min/1.73 m²)	66.25 ± 37.01	64.84 ± 21.01	t = 0.325	185	0.746
UACR (mg/g)	20.22 ± 56.96	11.83 ± 22.62	t = 1.347	181	0.180
Lipid parameters, mean ± SD					
HDL cholesterol (mg/dL)	44.57 ± 8.65	41.29 ± 5.60	t = 1.724	59	0.090
LDL cholesterol (mg/dL)	157.70 ± 69.58	196.32 ± 58.51	t = -4.241	196	<0.001
Triglycerides (mg/dL)	203.71 ± 59.51	221.39 ± 47.88	t = -2.303	194	0.022

Hypertension was the most frequently reported comorbidity among patients receiving ESM-25 and ESM-10 therapy, observed in 49.8% and 59.6% of patients, respectively. CKD was reported in 4.8% and 2.6% of patients receiving ESM-25 and ESM-10 therapy, respectively. Mean (SD) age was 55.55 (10.09) years in the ESM-25 group and 55.17 (8.97) years in the ESM-10 group. Mean (SD) body weight was 71.69 (11.86) kg and 72.00 (11.25) kg in the ESM-25 and ESM-10 groups, respectively. Utilization of ESM-25 therapy in patients with CAD was associated with mean (SD) HbA1c, FPG, and PPG levels of 9.47 (2.24), 160.12 (41.91) mg/dL, and 223.46 (65.31) mg/dL, respectively. Utilization of ESM-10 therapy was associated with mean (SD) HbA1c, FPG, and PPG levels of 9.05 (1.43), 159.61 (25.48) mg/dL, and 209.35 (49.33) mg/dL, respectively. Mean (SD) serum creatinine levels were 1.38 (1.09) mg/dL in the ESM-25 group and 1.25 (0.27) mg/dL in the ESM-10 group, whereas mean (SD) eGFR values were 66.25 (37.01) mL/min/1.73 m² and 64.84 (21.01) mL/min/1.73 m², respectively.

Statistically significant differences between ESM-25 and ESM-10 therapy were observed for healthcare provider specialty distribution (p < 0.0001), hypertension (p = 0.002), HbA1c (p = 0.026), RBG (p = 0.001), PPG (p = 0.044), LDL cholesterol (p < 0.0001), and triglyceride levels (p = 0.022), whereas age, gender distribution, body weight, FPG, serum creatinine, eGFR, UACR, and HDL cholesterol levels were comparable between the two dosage groups.

Triple drug therapy in IHD patients

Table 7 presents baseline clinical, biochemical, and cardiovascular characteristics associated with utilization of ESM triple therapy among patients with IHD. Among patients receiving ESM-25 therapy, 47.1% belonged to the 41-50 years age group, whereas 75.0% of patients receiving ESM-10 therapy were aged >50 years. A statistically significant association was observed between age group distribution and ESM triple therapy dosage groups (p = 0.013). Among healthcare provider specialties, diabetologists accounted for the highest proportion of ESM-25 prescriptions (47.1%), whereas cardiologists accounted for the highest proportion of ESM-10 prescriptions (41.7%). A statistically significant association was observed between healthcare provider specialty and ESM triple therapy dosage groups (p = 0.015).

**Table 7:** Clinical characteristics of patients with IHD receiving ESM triple therapy ESM: empagliflozin, sitagliptin, and metformin, CAD: coronary artery disease, IHD: ischemic heart disease, VHD: valvular heart disease, CVD: cardiovascular disease, CKD: chronic kidney disease, PAD: peripheral artery disease, RBG: random blood glucose, FPG: fasting plasma glucose, PPG: postprandial plasma glucose, eGFR: estimated glomerular filtration rate, UACR: urinary albumin-to-creatinine ratio, HDL: high-density lipoprotein, LDL: low-density lipoprotein.

Parameter	ESM-25 (n = 34)	ESM-10 (n = 24)	Statistical test	df	p-value
Age group, n (%)					
30-40 years	5 (14.7)	3 (12.5)	χ² = 8.737	2	0.013
41-50 years	16 (47.1)	3 (12.5)			
>50 years	13 (38.2)	18 (75.0)			
Healthcare provider specialty, n (%)					
Cardiologist	4 (11.8)	10 (41.7)	χ² = 10.493	4	0.015
Diabetologist	16 (47.1)	5 (20.8)			
Endocrinologist	6 (17.6)	1 (4.2)			
Nephrologist	0 (0.0)	0 (0.0)			
Physician	8 (23.5)	8 (33.3)			
Gender, n (%)					
Male	20 (58.8)	14 (58.3)	χ² = 0.001	1	0.970
Female	14 (41.2)	10 (41.7)			
Comorbidities, n (%)					
Hypertension	11 (32.4)	14 (58.3)	χ² = 6.346	5	0.175
CVD	16 (47.1)	7 (29.2)			
CKD	2 (5.9)	2 (8.3)			
PAD	1 (2.9)	1 (4.2)			
Other	0 (0.0)	0 (0.0)			
None	4 (11.8)	0 (0.0)			
Demographic parameters, mean ± SD					
Age (years)	54.12 ± 9.77	57.38 ± 9.27	t = -1.277	56	0.207
Weight (kg)	67.20 ± 11.61	71.14 ± 15.50	t = -0.601	15	0.557
Glycemic parameters					
HbA1c (%)	8.81 ± 1.14	7.63 ± 0.66	t = 2.521	19	0.021
RBG (mg/dL)	264.80 ± 68.59	217.33 ± 90.34	t = 0.849	6	0.428
FPG (mg/dL)	194.45 ± 52.66	146.67 ± 18.62	t = 2.124	15	0.051
PPG (mg/dL)	300.10 ± 75.38	206.83 ± 45.63	t = 2.724	14	0.016
Renal parameters, mean ± SD					
Serum creatinine (mg/dL)	1.42 ± 0.47	3.55 ± 3.61	t = -0.830	2	0.494
eGFR (mL/min/1.73 m²)	57.00 ± 38.18	49.00 ± 53.74	t = 0.172	2	0.880
UACR (mg/g)	187.50 ± 229.81	24.10 ± 25.31	t = 0.999	2	0.423
Lipid parameters, mean ± SD					
HDL cholesterol (mg/dL)	-	46.00 ± 4.24	-	-	-
LDL cholesterol (mg/dL)	91.67 ± 40.72	77.33 ± 65.29	t = 0.323	4	0.763
Triglycerides (mg/dL)	192.40 ± 107.64	195.00 ± 43.00	t = −0.039	6	0.970

Male patients accounted for 58.8% and 58.3% of patients receiving ESM-25 and ESM-10 therapy, respectively. Hypertension was reported in 32.4% of patients receiving ESM-25 therapy and 58.3% of patients receiving ESM-10 therapy, whereas CVD was reported in 47.1% and 29.2% of patients, respectively. Mean (SD) age was 54.12 (9.77) years in the ESM-25 group and 57.38 (9.27) years in the ESM-10 group. Mean (SD) body weight was 67.20 (11.61) kg and 71.14 (15.50) kg in the ESM-25 and ESM-10 groups, respectively.

Utilization of ESM-25 therapy in patients with IHD was associated with mean (SD) HbA1c, FPG, and PPG levels of 8.81 (1.14)%, 194.45 (52.66) mg/dL, and 300.10 (75.38) mg/dL, respectively. Utilization of ESM-10 therapy was associated with mean (SD) HbA1c, FPG, and PPG levels of 7.63 (0.66)%, 146.67 (18.62) mg/dL, and 206.83 (45.63) mg/dL, respectively. Statistically significant differences between ESM-25 and ESM-10 therapy were observed for HbA1c (p = 0.021) and PPG levels (p = 0.016), whereas age, body weight, RBG, renal parameters, and lipid parameters were comparable between the two dosage groups.

Triple drug therapy in heart failure patients

Table 8 presents baseline clinical, biochemical, and cardiovascular characteristics associated with utilization of ESM triple therapy among patients with heart failure. Among patients receiving ESM-25 therapy, 64.5% were aged >50 years, whereas 58.4% of patients receiving ESM-10 therapy belonged to the same age category. Male patients accounted for 60.4% and 69.9% of patients receiving ESM-25 and ESM-10 therapy, respectively.

**Table 8:** Clinical characteristics of patients with heart failure receiving ESM triple therapy ESM: empagliflozin, sitagliptin, and metformin, CAD: coronary artery disease, IHD: ischemic heart disease, VHD: valvular heart disease, CVD: cardiovascular disease, CKD: chronic kidney disease, PAD: peripheral artery disease, RBG: random blood glucose, FPG: fasting plasma glucose, PPG: postprandial plasma glucose, eGFR: estimated glomerular filtration rate, UACR: urinary albumin-to-creatinine ratio, HDL: high-density lipoprotein, LDL: low-density lipoprotein.

Parameter	ESM-25 (n = 217)	ESM-10 (n = 219)	Statistical test	df	p-value
Age group, n (%)					
30-40 years	19 (8.8)	28 (12.8)	χ² = 2.458	2	0.293
41-50 years	58 (26.7)	63 (28.8)			
>50 years	140 (64.5)	128 (58.4)			
Healthcare provider specialty, n (%)					
Cardiologist	31 (14.3)	89 (40.6)	χ² = 130.841	4	<0.001
Diabetologist	127 (58.5)	20 (9.1)			
Endocrinologist	29 (13.4)	27 (12.3)			
Nephrologist	0 (0.0)	0 (0.0)			
Physician	30 (13.8)	83 (37.9)			
Gender, n (%)					
Male	131 (60.4)	153 (69.9)	χ² = 4.327	1	0.038
Female	86 (39.6)	66 (30.1)			
Comorbidities, n (%)					
Hypertension	84 (38.7)	42 (19.2)	χ² = 37.008	5	<0.001
CVD	98 (45.2)	132 (60.3)			
CKD	20 (9.2)	21 (9.6)			
PAD	1 (0.5)	1 (0.5)			
Other	8 (3.7)	0 (0.0)			
None	6 (2.8)	23 (10.5)			
Demographic parameters, mean ± SD					
Age (years)	54.12 ± 9.16	54.78 ± 9.55	t = -0.738	434	0.461
Weight (kg)	67.33 ± 13.05	67.12 ± 12.26	t = 0.130	259	0.896
Glycemic parameters, mean ± SD					
HbA1c (%)	8.61 ± 1.18	8.69 ± 1.49	t = -0.488	299	0.626
RBG (mg/dL)	216.70 ± 66.17	222.72 ± 53.45	t = -0.572	132	0.569
FPG (mg/dL)	166.69 ± 34.31	157.44 ± 27.44	t = 2.441	264	0.015
PPG (mg/dL)	251.21 ± 60.06	221.45 ± 45.66	t = 4.564	261	<0.001
Renal parameters, mean ± SD					
Serum creatinine (mg/dL)	0.99 ± 0.28	1.36 ± 0.67	t = -3.223	102	0.002
eGFR (mL/min/1.73 m²)	89.92 ± 38.31	65.09 ± 21.72	t = 4.244	103	<0.001
UACR (mg/g)	63.13 ± 105.80	16.17 ± 20.75	t = 3.494	98	0.001
Lipid parameters, mean ± SD					
HDL cholesterol (mg/dL)	45.54 ± 7.07	42.55 ± 5.69	t = 1.738	53	0.088
LDL cholesterol (mg/dL)	109.03 ± 48.89	184.68 ± 77.01	t = -6.387	127	<0.001
Triglycerides (mg/dL)	170.96 ± 55.30	214.39 ± 49.39	t = -4.512	120	<0.001

Among healthcare provider specialties, diabetologists accounted for the highest proportion of ESM-25 prescriptions (58.5%), whereas cardiologists accounted for the highest proportion of ESM-10 prescriptions (40.6%). A statistically significant association was observed between healthcare provider specialty and ESM triple therapy dosage groups (p < 0.0001). Hypertension was reported in 38.7% of patients receiving ESM-25 therapy and 19.2% of patients receiving ESM-10 therapy, whereas CKD was reported in 9.2% and 9.6% of patients, respectively.

Mean (SD) age was 54.12 (9.16) years in the ESM-25 group and 54.78 (9.55) years in the ESM-10 group. Mean (SD) body weight was 67.33 (13.05) kg and 67.12 (12.26) kg in the ESM-25 and ESM-10 groups, respectively. Utilization of ESM-25 therapy in patients with heart failure was associated with mean (SD) HbA1c, FPG, and PPG levels of 8.61 (1.18), 166.69 (34.31) mg/dL, and 251.21 (60.06) mg/dL, respectively. Utilization of ESM-10 therapy in patients with heart failure was associated with mean (SD) HbA1c, FPG, and PPG levels of 8.69 (1.49), 157.44 (27.44) mg/dL, and 221.45 (45.66) mg/dL, respectively.

Mean (SD) serum creatinine levels were 0.99 (0.28) mg/dL in the ESM-25 group and 1.36 (0.67) mg/dL in the ESM-10 group, whereas mean (SD) eGFR values were 89.92 (38.31) mL/min/1.73 m² and 65.09 (21.72) mL/min/1.73 m², respectively. Statistically significant differences between ESM-25 and ESM-10 therapy were observed for healthcare provider specialty distribution (p < 0.0001), gender distribution (p = 0.038), hypertension (p < 0.0001), FPG (p = 0.015), PPG (p < 0.0001), serum creatinine (p = 0.002), eGFR (p < 0.0001), UACR (p = 0.001), LDL cholesterol (p<0.001), and triglyceride levels (p<0.0001), whereas age, body weight, HbA1c, RBG, and HDL cholesterol levels were comparable between the two dosage groups.

Triple drug therapy in VHD patients

Table 9 presents baseline clinical characteristics associated with utilization of ESM triple therapy among patients with VHD. Among patients receiving ESM-25 therapy, 93.8% were aged >50 years, whereas 50.0% of patients receiving ESM-10 therapy belonged to the same age category. A statistically significant association was observed between age group distribution and ESM triple therapy dosage groups (p = 0.049). Among healthcare provider specialties, diabetologists accounted for the highest proportion of ESM triple therapy prescriptions in both the ESM-25 (75.0%) and ESM-10 (66.7%) groups. Male patients accounted for 62.5% and 66.7% of patients receiving ESM-25 and ESM-10 therapy, respectively. Hypertension was the most commonly reported comorbidity among patients with VHD receiving ESM-25 and ESM-10 therapy, observed in 43.8% and 33.3% of patients, respectively. The observed differences in clinical characteristics and prescribing patterns primarily reflect individualized therapeutic decision-making in routine clinical practice.

**Table 9:** Clinical characteristics of patients with VHD receiving ESM triple therapy ESM: empagliflozin, sitagliptin, and metformin, VHD: valvular heart disease, CVD: cardiovascular disease, CKD: chronic kidney disease, PAD: peripheral artery disease.

Parameter	ESM-25 (n = 16)	ESM-10 (n = 6)	Statistical test	df	p-value
Age group, n (%)					
30-40 years	0 (0.0)	1 (16.7)	χ² = 6.035	2	0.049
41-50 years	1 (6.3)	2 (33.3)			
>50 years	15 (93.8)	3 (50.0)			
Healthcare provider specialty, n (%)					
Cardiologist	0 (0.0)	1 (16.7)	χ² = 3.094	4	0.377
Diabetologist	12 (75.0)	4 (66.7)			
Endocrinologist	1 (6.3)	0 (0.0)			
Nephrologist	0 (0.0)	0 (0.0)			
Physician	3 (18.8)	1 (16.7)			
Gender, n (%)					
Male	10 (62.5)	4 (66.7)	χ² = 0.033	1	0.631
Female	6 (37.5)	2 (33.3)			
Comorbidities, n (%)					
Hypertension	7 (43.8)	2 (33.3)	χ² = 3.234	5	0.664
CVD	2 (12.5)	2 (33.3)			
CKD	3 (18.8)	0 (0.0)			
PAD	2 (12.5)	1 (16.7)			
Other	1 (6.3)	0 (0.0)			
None	1 (6.3)	1 (16.7)			

## Discussion

The present multicentric real-world evidence study describes prescribing patterns and clinical characteristics among Indian patients with T2DM receiving ESM-based triple FDC therapy in routine clinical practice. The study provides descriptive information on demographic characteristics, glycemic parameters, renal and lipid profiles, documented comorbidities, and prescriber specialties across the ESM-25 and ESM-10 groups. The demographic profile observed in the present study is consistent with the known epidemiological characteristics of T2DM in India, where the disease predominantly affects middle-aged and older adults with a high burden of associated cardiometabolic risk factors. The predominance of older patients describes the demographic profile of the study population; however, disease duration and treatment intensification were not assessed in this study, and no causal inference regarding the reasons for treatment selection can be made. Male predominance observed across the study population is also consistent with previous Indian real-world studies reporting higher healthcare utilization among men with T2DM. Physicians and diabetologists accounted for the majority of documented ESM prescriptions in the study population. This finding describes the distribution of prescribing across the specialties represented in the available records but should not be interpreted as evidence of widespread or national utilization of ESM therapy. Furthermore, the variability in prescribing patterns across healthcare specialties reflects evolving implementation of individualized treatment strategies aligned with contemporary diabetes management guidelines [4,5] and should not be interpreted as evidence of prescribing rationale or changing treatment preferences. Similar prescribing patterns have been reported in previous real-world observational studies evaluating empagliflozin-based combination therapies among patients with T2DM and associated cardiometabolic conditions [14,15].

In routine clinical practice, treatment intensification strategies are frequently guided by baseline glycemic burden and associated cardiometabolic risk factors [4]. Patients receiving ESM-25 therapy generally demonstrated comparatively higher glycemic parameters than those receiving ESM-10 therapy, including HbA1c (8.80% vs 8.68%), FPG (182.38 mg/dL vs 163.96 mg/dL), and PPG (247.99 mg/dL vs 216.54 mg/dL). Although the differences in FPG and PPG were statistically significant, their magnitude should be interpreted in the context of the cross-sectional design and available data. The HbA1c difference was only 0.12 percentage points and had a very small effect size (Cohen's d = 0.079), indicating that statistical significance should not be equated with clinical importance. These findings describe differences in glycemic characteristics between patients receiving the two prescribed ESM strengths and do not establish treatment intensification or comparative treatment effects. The complementary glucose-lowering mechanisms of ESM provide pharmacological context for the combination but were not evaluated as treatment effects in the present study.

Body weight was comparable between the two dose groups. However, the study did not collect information on the factors considered by clinicians when selecting the prescribed dose; therefore, the relative contribution of glycemic status, comorbidity burden, body weight, renal function, prior therapy, drug availability, cost, patient preference, or formulary considerations to dose selection cannot be determined. The observed differences in clinical characteristics between ESM-25 and ESM-10 should therefore be interpreted as descriptive associations rather than evidence of treatment intensification, prescribing rationale, or dose preference. Previous real-world studies evaluating empagliflozin-based combination therapies provide relevant external context, but their findings cannot establish the reasons underlying dose selection in the present study [4,16].

Contemporary diabetes management guidelines increasingly emphasize early identification and management of cardiovascular and renal risk in T2DM, with treatment selection extending beyond glycemic control alone [4,5]. Current recommendations advocate preferential use of empagliflozin-based therapies in individuals with established cardiovascular disease, heart failure, or CKD because of their proven cardiorenal protective benefits [4,16]. In this context, the substantial burden of cardiovascular and renal comorbidities observed in the present study further highlights the complex clinical profile frequently encountered in routine practice.

The relatively low proportion of documented hypertension, CVD, and CKD may partly reflect under-recording or incomplete documentation in the retrospective medical records. As comorbidities were identified from available source records rather than through independent clinical reassessment, the reported proportions should be interpreted as documented comorbidity prevalence and may underestimate the true burden of comorbid conditions in this population. Hypertension was the most frequently reported comorbidity, affecting 6.2% (n = 797) of the overall study population, followed by CVD in 4.1% (n = 526) and CKD in 1.1% (n = 145). Subgroup analyses described the demographic, clinical, laboratory, and prescribing characteristics of patients with documented CAD, IHD, heart failure, and CKD who were already receiving ESM therapy. These subgroup findings should be interpreted as descriptive associations and not as evidence of treatment preference, effectiveness, or clinical benefit. Greater representation of cardiologists among prescribers in some cardiovascular subgroups describes the specialty distribution observed in the available records. The study did not assess the clinical rationale underlying these prescribing patterns. Previous cardiovascular outcome trials and real-world observational studies have demonstrated cardiovascular and renal benefits of empagliflozin in appropriately studied populations [9,17]. These external findings provide clinical context but do not represent outcomes evaluated in the present study.

Cardiovascular comorbidities remain a major determinant of therapeutic decision-making in patients with longstanding T2DM, particularly among those with established atherosclerotic and cardiorenal disease. In the present study, subgroup analyses demonstrated distinct prescribing trends across patients with CAD, IHD, and heart failure. Higher utilization of ESM-25 therapy was generally observed among individuals with greater glycemic and metabolic burden, whereas ESM-10 therapy was more frequently prescribed in patients with comparatively higher cardiovascular comorbidity burden. Cardiologists contributed substantially to prescribing decisions within cardiovascular subgroups, reflecting the increasing incorporation of SGLT2 inhibitor-based therapies into contemporary cardiovascular risk management strategies. Patients with heart failure receiving ESM therapy additionally demonstrated substantial associated comorbidity burden and older age distribution, highlighting the complex clinical profile frequently encountered in routine practice. These descriptive findings provide clinical context for previous evidence supporting cardiovascular and renal benefits of empagliflozin in appropriately studied populations [18]. However, cardiovascular or renal outcomes were not evaluated in the present study, and therefore these established benefits cannot be inferred as outcomes of the ESM triple FDC from the current data. Furthermore, the observed lipid and renal parameter differences across cardiovascular subgroups reinforce the importance of comprehensive cardiometabolic risk assessment while selecting individualized antidiabetic therapy in real-world clinical settings [4].

The observed differences in renal parameters likely reflect baseline renal risk profiles among patients selected for different empagliflozin dosage strengths rather than comparative effects of the therapies, given the retrospective cross-sectional study design [4,19]. Dedicated CKD subgroup analyses further showed extensive utilization of ESM therapy among patients with underlying renal impairment. These findings describe the clinical profiles of patients with CKD receiving ESM therapy in routine practice; however, the study did not assess clinicians’ reasons for prescribing or renal outcomes. The observed prescribing pattern indicates that management of patients with CKD receiving ESM therapy was predominantly undertaken by diabetologists, physicians, endocrinologists, and cardiologists, which may reflect routine management of T2DM patients with early-stage CKD in diabetes-focused clinical settings. These findings are particularly relevant in view of current guideline recommendations advocating early incorporation of SGLT2 inhibitors in patients with T2DM and CKD to reduce progression of renal dysfunction and associated cardiovascular complications. In addition to its glucose-lowering effects, empagliflozin has demonstrated favorable renal hemodynamic and anti-inflammatory effects, which may help slow CKD progression and reduce albuminuria. Similar renoprotective benefits associated with empagliflozin have been consistently demonstrated in major clinical trials and real-world studies evaluating SGLT2 inhibitor-based therapies among patients with T2DM and CKD [14,17,20-22].

The lipid profile findings further reflect the metabolic complexity commonly observed in patients with T2DM receiving combination antidiabetic therapy in routine clinical practice. Patients receiving ESM-25 therapy demonstrated comparatively lower LDL cholesterol and triglyceride levels than those receiving ESM-10 therapy, whereas HDL cholesterol levels remained broadly comparable between the two groups. Although dyslipidemia in T2DM is multifactorial and influenced by several patient-related variables, these observations may partly reflect more intensive metabolic management among individuals receiving higher-dose empagliflozin therapy. Emerging evidence suggests that SGLT2 inhibitors may exert modest favorable effects on lipid metabolism through improvements in insulin resistance, weight reduction, and altered substrate utilization [21,23]. Furthermore, the integrated use of SGLT2 inhibitor-based therapies in patients with elevated cardiometabolic risk is increasingly supported by contemporary diabetes management strategies emphasizing comprehensive risk reduction beyond glycemic control alone. Similar improvements in metabolic and cardiometabolic parameters have been reported in real-world studies evaluating empagliflozin-based combination therapies among patients with T2DM [24,25]. The addition of sitagliptin and metformin to empagliflozin-based therapy may further support broader metabolic stabilization through complementary effects on insulin resistance, postprandial glucose excursions, and overall glycemic variability.

Overall, the present study provides descriptive real-world information on the clinical characteristics and prescribing patterns of patients receiving ESM-based triple FDC therapy. Differences between ESM-25 and ESM-10 were observed across selected glycemic, renal, lipid, demographic, and prescriber characteristics, as well as within selected cardiovascular and renal subgroups. However, these findings describe associations among patients already receiving ESM therapy and do not establish comparative effectiveness, treatment superiority, treatment intensification, or the reasons underlying dose selection. The cardiovascular and renal benefits of empagliflozin demonstrated in previous clinical trials provide relevant context but were not evaluated as outcomes in this study.

Strengths

The major strengths of the present study include its large multicentric real-world cohort of 12,843 patients and its evaluation of ESM-FDC prescribing patterns in routine clinical practice across healthcare centers in India. The study provides a broad clinical characterization of patients receiving ESM-25 and ESM-10, including demographic characteristics, glycemic parameters, renal and lipid profiles, comorbidities, and healthcare provider specialty. The inclusion of cardiovascular and renal comorbidity subgroups provides additional descriptive information regarding the clinical profiles of patients receiving ESM therapy in routine practice. The study also reports between-group effect estimates and 95% confidence intervals for major continuous variables, supporting a more informative assessment of the magnitude and precision of observed differences.

Limitations

Despite the strengths of the large multicentric real-world dataset, several limitations should be considered. The retrospective cross-sectional design precludes assessment of temporal relationships, treatment effectiveness, adherence, and longitudinal outcomes and does not allow causal inference. As patients were already receiving ESM-25 or ESM-10, differences between groups may be affected by confounding by indication and unmeasured factors, and the rationale for dose selection could not be determined. Potentially relevant factors, including prior therapy, patient preference, drug availability, cost, and formulary considerations, were not consistently available, and no adjustment for potential confounders was performed. Laboratory data were available only for a subset of patients, resulting in variable sample sizes and potential selection bias with available-case analysis. A standardized patient-level index date and predefined laboratory time window were also not consistently available. Very small sample sizes in selected CKD, heart failure, IHD, and VHD subgroup analyses, particularly for laboratory parameters, limit the stability of these findings; use of parametric testing for highly variable measures such as UACR may further affect robustness. Multiple comparisons were performed without formal multiplicity adjustment, increasing the possibility of chance findings. Comorbidities were based on documented diagnoses without independent reassessment, and the low documented prevalence of hypertension, CVD, and CKD may reflect incomplete ascertainment or under-recording. Therefore, differences between ESM-25 and ESM-10 should be interpreted as descriptive associations rather than evidence of treatment superiority, treatment intensification, or comparative efficacy.

## Conclusions

The present multicentric real-world evidence study describes prescribing patterns and clinical characteristics among Indian patients with T2DM receiving ESM-based triple FDC therapy. Patients receiving ESM-25 and ESM-10 demonstrated differences in selected glycemic, renal, lipid, and prescribing characteristics. These findings represent descriptive associations within patients already receiving ESM therapy and should not be interpreted as evidence of comparative treatment effectiveness, treatment intensification, dose superiority, or the clinical rationale underlying dose selection. The study also describes the clinical characteristics of patients receiving ESM therapy across selected cardiovascular and renal comorbidity subgroups. Overall, the findings provide descriptive real-world information regarding the clinical profiles and prescribing patterns associated with the two empagliflozin strengths in Indian clinical practice.
